# E3 ubiquitin ligase NEDD4 inhibits PEDV infection through ubiquitination and degradation of the viral primase NSP8

**DOI:** 10.1128/jvi.02156-25

**Published:** 2026-03-30

**Authors:** Xuyang Guo, Xiaojing Zhao, Jianyu He, Yuchen Lu, Sijia Jin, Zhiqian Ma, Zhiwei Li, Zifang Zheng, Yang Li, Yingtong Feng, Lele Xu, Xiao Liu, Jianwu Zhang, Zhanyong Wei, Haixue Zheng, Shuqi Xiao

**Affiliations:** 1 State Key Laboratory of Animal Disease Control and Prevention, College of Veterinary Medicine, Lanzhou University, Lanzhou Veterinary Research Institute, Chinese Academy of Agricultural Sciences, Lanzhou, China; 2 Gansu Province Research Center for Basic Disciplines of Pathogen Biology, Lanzhou Veterinary Research Institute, Chinese Academy of Agricultural Sciences 111658, Lanzhou, China; 3 Ministry of Education Key Laboratory for Animal Pathogens and Biosafety, Henan Agricultural University 70573, Zhengzhou, China; Loyola University Chicago - Health Sciences Campus, Maywood, Illinois, USA

**Keywords:** PEDV, NEDD4, NSP8, ubiquitin, NDP52

## Abstract

Porcine epidemic diarrhea virus (PEDV), a highly lethal alpha coronavirus, primarily infects piglet intestines, yet the role of the intestinal homeostasis regulator NEDD4—a HECT-family E3 ubiquitin ligase—in PEDV infection was previously unknown. This study reveals that NEDD4 inhibits PEDV infection by mediating K63-linked ubiquitination of the viral primase NSP8, targeting a conserved lysine (K170) within an NEDD4-recognized SPP motif. The C2 domain of NEDD4 is critical for this antiviral activity, as deletion of its RDDFLGQVDVPLYPLPT exon abolishes PEDV suppression and disrupts NSP8 binding, with AlphaFold3 structural modeling showing this deletion induces conformational changes at the C2-WW interdomain junction. PEDV infection upregulates NEDD4 expression *in vivo* and *in vitro*, and functional experiments confirm that NEDD4 overexpression suppresses viral replication while knockout enhances it. Ubiquitinated NSP8 is degraded via both NDP52-dependent selective autophagy and the ubiquitin-proteasome system, with NEDD4 further amplifying autophagy through beclin-1 upregulation. These findings not only elucidate a host antiviral mechanism whereby NEDD4 restricts PEDV infection through dual degradation of NSP8 but also highlight the therapeutic potential of targeting the NEDD4-NSP8 axis to combat PEDV infection.

**IMPORTANCE:**

Porcine epidemic diarrhea virus (PEDV) is a highly lethal alpha coronavirus that causes devastating economic losses in the swine industry worldwide, yet effective control measures remain limited due to an incomplete understanding of host antiviral mechanisms. This study narrows this critical knowledge gap by identifying NEDD4 as a pivotal host defense factor that suppresses PEDV replication through a novel dual-pathway mechanism: mediating K63-linked ubiquitination of the viral primase NSP8 at a conserved motif K170 to trigger its degradation via both selective autophagy and the proteasome system while enhancing antiviral autophagy through beclin-1 upregulation. Our work establishes the NEDD4-NSP8 axis as a promising therapeutic target for the development of much-needed antiviral strategies to combat PEDV and related coronaviral infections.

## INTRODUCTION

Porcine epidemic diarrhea virus (PEDV), a single-stranded positive-sense RNA virus, belongs to the genus alpha coronavirus in the family Coronaviridae. A highly virulent PEDV emerged in China in 2010 that then spread rapidly to many countries and regions, leading to significant economic losses to the swine industry worldwide (1). In recent years, new variants of PEDV have been generated through recombination and mutation, which further aggravates the epidemic of PEDV. It has been reported that PEDV originates from bats and possesses cross-species transmission (2). PEDV primarily targets the small intestinal tissue of pigs, with the infection process centered on replication and spread within this organ. The virus poses a particularly severe threat to newborn piglets, manifesting clinically as characteristic watery diarrhea, persistent vomiting, and severe dehydration, with mortality rates reaching up to 100% in infected piglets (3). Due to their underdeveloped digestive systems and weak intestinal mucosal immune barriers, newborn piglets rely mainly on maternal antibodies for passive immune protection. This makes PEDV infection highly destructive to intestinal microecological balance, leading to severe pathological damage. Notably, PEDV possesses the high mutation propensity inherent to coronaviruses, and this characteristic, combined with its unique infection mechanism, means that effective control of the virus heavily depends on strict biosecurity measures (4). However, for many developing countries, establishing a comprehensive biosecurity system remains a significant challenge. Meanwhile, the global spread of PEDV provides favorable conditions for viral mutation, creating a vicious cycle of epidemic-driven evolution that continuously exacerbates the threat PEDV poses to the swine industry, presenting a formidable challenge for disease prevention and control (5).

Although PEDV exhibits a high capacity for mutation and recombination, its core components—particularly those essential for viral replication—are generally highly conserved. Understanding how host cells target these conserved core elements to combat viral infection is crucial for developing broad-spectrum antiviral drugs to counter the threat posed by PEDV variants. The core replication components of PEDV include RNA-dependent RNA polymerase Nsp12, helicase NSP13, exonuclease NSP14, as well as NSP7 and NSP8, which stabilize the viral genomic RNA. Notably, NSP8 also functions as a primase during viral genome RNA replication (6). Recent studies have shown that SARS-CoV-2 NSP8 promotes viral replication by interfering with host-cell autophagy and immune responses (7, 8), while host cells can also employ multiple mechanisms to degrade coronavirus NSP8 and inhibit infection (9, 10). However, the specific molecular mechanisms by which host cells degrade PEDV NSP8 remain unclear and warrant further investigation.

Neural precursor cell expressed developmentally downregulated protein 4 (NEDD4) belongs to the HECT ubiquitin E3 ligase family, which is evolutionarily conserved from yeast to mammals (11). NEDD4 regulates several cellular processes, including cargo sorting, intracellular trafficking, and degradation of a large number of proteins in multiple cellular compartments by the ubiquitination of its specific substrates (12). NEDD4 is a critical regulator of intestinal homeostasis, which modulates intestinal epithelial cell function and stem cell activation through mechanisms, including the WNT signaling pathway, ferroptosis, and calcium ion flux, thereby maintaining the intestinal homeostasis. However, the interplay between NEDD4 and PEDV infection remains unclear. NEDD4 not only regulates viral infection by modulating host cell functions but also directly targets viral proteins to control their replication process. NEDD4 is deeply involved in all stages of viral infection, especially important in the virus budding (13–15). It has been reported that Adenovirus protein VI recruits NEDD4 to bind and ubiquitylate protein VI which facilitates Adenovirus rapid entry by microtubule-dependent intracellular movement (16). Besides, NEDD4 promotes influenza virus infection by decreasing levels of the antiviral protein interferon-induced transmembrane protein 3 (17). During virus budding, NEDD4 not only directly interacts with the viral protein PPxY domain but also cooperates with Tsg101 to regulate viral particle maturation (13). Diametrically opposed to this, NEDD4 is also a sharp tool to inhibit viral infection by targeting and degrading viral proteins (18).

A recent report showed HECT E3 ligases entanglement in SARS-CoV-2 infection by interacting with Spike protein PPxY domain and ubiquitylating the SARS-CoV-2 Spike protein (19). However, the PEDV Spike protein does not have a PPxY domain. Based on the crucial regulatory role of NEDD4 in intestinal homeostasis, we investigated the potential biological functions of NEDD4 during PEDV infection. During PEDV infection *in vivo* and *in vitro*, NEDD4 expression is significantly upregulated. NEDD4 binds and ubiquitinates the viral protein NSP8, promoting its degradation via the UPS and NDP52-mediated selective autophagy, thereby suppressing viral infection. These findings reveal a novel host regulatory mechanism for NSP8 and identify NEDD4 as a potential target to enhance antiviral responses.

## RESULTS

### NEDD4 was upregulated by PEDV infection *in vivo* and *in vitro*

**Fig 1:**
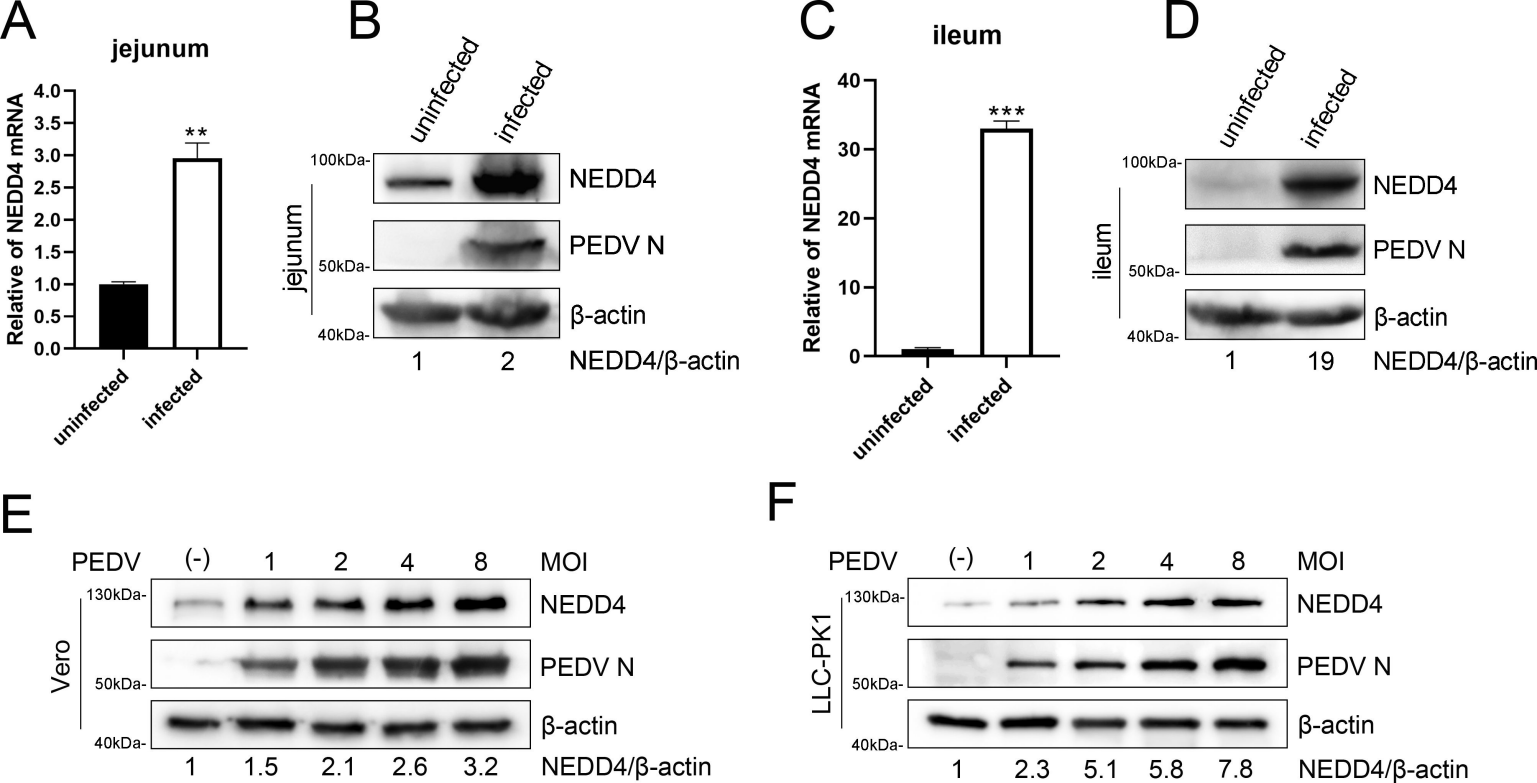
PEDV infection upregulates NEDD4 expression. (**A–D**) RT-qPCR and immunoblot analysis of NEDD4 expression in jejunal and ileal tissues from PEDV-infected deceased piglets and healthy controls. (**E, F**) immunoblot analysis of NEDD4 in Vero and LLC-PK1 cells following PEDV infection at increasing MOIs. A single asterisk (*) signifies a *P*-value less than 0.05, (**) signifies *P*-value less than 0.01, and (***) signifies *P*-value less than 0.001.

Given that NEDD4 regulates the homeostasis of intestinal epithelial cells and intestinal stem cells (20), jejunal and ileal tissues from piglets that died of PEDV infection were collected to analyze NEDD4 expression changes for investigating its role in PEDV infection. Both mRNA and protein expression analyses demonstrated significantly elevated NEDD4 levels in PEDV-infected jejunum and ileum tissues (Fig. 1A through D). We subsequently analyzed the impact of PEDV infection on NEDD4 expression in Vero and LLC-PK1 cells, two commonly used cell lines for PEDV *in vitro* cultivation. Vero and LLC-PK1 cells were infected with PEDV at an increased MOI and harvested at 24 h. The Western blot results showed that the expression level of NEDD4 in both Vero and LLC-PK1 cells increased with PEDV infection (Fig. 1E and F). Collectively, these results demonstrate that PEDV infection induces NEDD4 expression both *in vivo* and *in vitro*.

### Knockout of NEDD4 enhances PEDV infection

**Fig 2:**
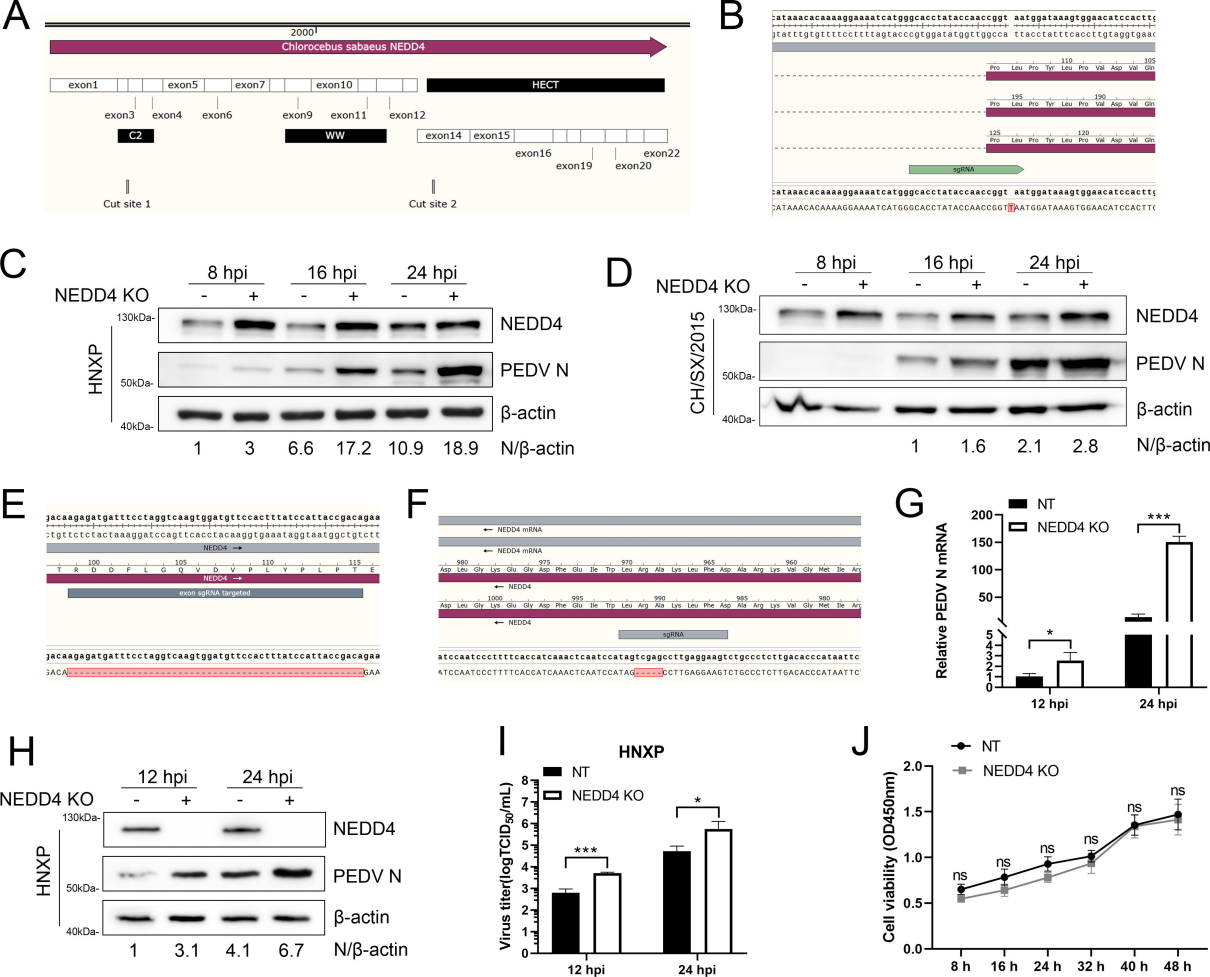
Knockout of NEDD4 enhances PEDV infection. (**A**) Schematic diagram of NEDD4 C2 and HECT domain knockout sites. (**B**) Sequencing alignment of NEDD4 genomic knockout sites in single-cell subclones with C2 domain site knockout. (**C and D**) Immunoblot analysis of PEDV replication levels in NEDD4 KO (targeting C2 domain) and NT cells. (**E**) Comparative analysis of NEDD4 mRNA from NEDD4 KO cells. (**F**) Sequencing alignment of NEDD4 genomic knockout sites in single-cell subclones with HECT domain site knockout. (**G and H**) RT-qPCR and immunoblot analysis of PEDV replication levels in NEDD4 KO (targeting HECT domain) and NT cells. (**I**) Virus titer determination of PEDV replication levels in NEDD4 KO (targeting HECT domain) and NT cells. (**J**) The cell viability of NEDD4 HECT KO cells in comparison with NT cells. A single asterisk (*) signifies a *P*-value less than 0.05, (**) signifies *P*-value less than 0.01, and (***) signifies *P*-value less than 0.001.

NEDD4 possesses three functional domains: C2, WW, and HECT. The C2 domain is a Ca^2+^-dependent binding domain that primarily directs NEDD4 to membrane compartments (21). The WW domains mediate protein–protein interactions by recognizing Pro-rich motifs, while the HECT domain possesses ubiquitin ligase activity (Fig. 2A). To determine the role of NEDD4 in PEDV replication, we generated a frameshift knockout in Vero cells using single-guide RNA (sgRNA) targeting the preceding exon of all NEDD4 variant genes (Fig. 2A). After single-cell subcloning, we got one NEDD4 frameshift knockout clone, confirmed by Sanger sequencing (Fig. 2B). Then, the cells were infected with PEDV classic strain CH/SX/2015 or prevalent strain HNXP at an MOI of 1 for 8, 16, and 24 h. Protein samples were subjected to immunoblotting analysis, which revealed that genetic ablation of NEDD4 consistently suppressed PEDV N protein production across all examined time courses (Fig. 2C and D). Contrary to expectations, the knockout procedure resulted in compensatory upregulation of NEDD4 expression rather than its elimination (Fig. 2C and D). We speculated that there may exist a partial deficiency in the gene that is critical for the function of NEDD4, leading to the compensatory expression of NEDD4. Subsequently, NEDD4 mRNA in the knockout cell line was amplified and sequenced to verify the genetic modification. As expected, the exon 3 sgRNA-targeted region was deleted, and a nonexistent NEDD4 mRNA was still formed by connecting back and forth exon (Fig. 2E). A new sgRNA targeting NEDD4 HECT domain was synthesized, and single-cell subclone was performed (Fig. 2A), sequencing results confirmed successful establishment of a cell line carrying a 5-nucleotide deletion in the HECT domain (Fig. 2F). The cells were then infected with PEDV HNXP at an MOI of 1 for 12 or 24 h, harvested cells were analyzed by quantitative real-time PCR and immunoblotting to detect changes in PEDV N mRNA and protein expression levels, respectively. Both PEDV N mRNA and protein levels were obviously increased in the NEDD4 HECT KO cells than in NT cells (Fig. 2G and H). Consistent with this, measurement of viral titers demonstrated a marked increase in progeny virus generation upon NEDD4 knockout (Fig. 2I). Cell viability, detected using a cell counting kit (CCK) assay, illustrated that the knockout of NEDD4 did not affect the growth of Vero cells (Fig. 2J). Together, these results indicate that NEDD4 plays a role in the inhibition of PEDV replication.

### Overexpression of NEDD4 inhibits PEDV infection

**Fig 3:**
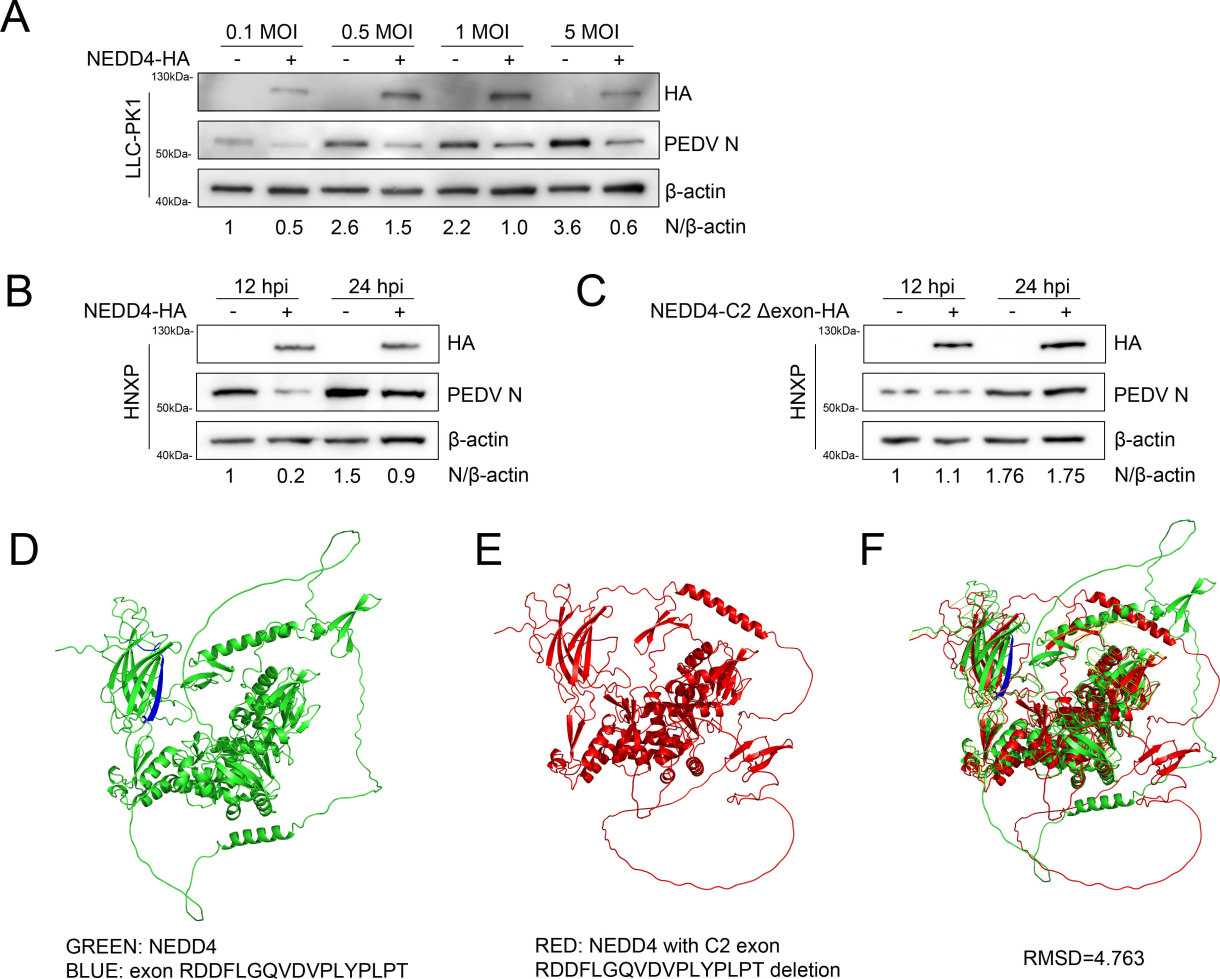
Overexpression of NEDD4 inhibits PEDV infection. (**A**) Immunoblot analysis of PEDV replication levels in NEDD4-overexpressing LLC-PK1 cells infected with increasing MOIs of PEDV. (**B**) Immunoblot analysis of PEDV replication levels in NEDD4-overexpressing Vero cells infected with PEDV for 12 and 24 h. (**C**) Immunoblot analysis of PEDV replication levels in NEDD4 C2 deletion-overexpressing Vero cells infected with PEDV for 12 and 24 h. (**D and E**) Structural modeling of NEDD4 and its C2 domain single exon deletion variant using AlphaFold3. (**F**) Structural alignment and analysis of NEDD4 and its C2 domain single exon deletion variant using PyMol. RMSD<1. An RMSD value of less than 1 indicates highly similar structures, an RMSD value of greater than 1 suggests certain differences, and an RMSD value exceeding 2 signifies significant differences.

To further confirm the negative role of NEDD4 in PEDV replication, the porcine NEDD4 proteins were overexpressed in LLC-PK1 cells for 24 h, and the cells were then infected with PEDV HNXP in a dose-dependent manner. The infected cell cultures were collected and assayed for the PEDV viral loads by immunoblotting. As expected, the PEDV N proteins were significantly decreased in the LLC-PK1 cells transfected with the NEDD4-HA vector under different amounts of PEDV (Fig. 3A). Subsequently, NEDD4-HA was further transfected into Vero cells for 24 h, followed by infection with PEDV at an MOI of 1. Cell lysates were collected at 12 and 24 h for Western blot analysis. Results indicated that NEDD4 overexpression significantly decreased PEDV N protein expression compared to control groups (Fig. 3B). Then, to further confirm the impact of the abnormal variant caused by NEDD4 C2 knockout on PEDV infection, Vero cells transfected with the NEDD4 C2 single-exon deletion plasmid (same exon as knockout) were infected with PEDV at 24 h. Western blot results demonstrated that partial C2 domain deletion eliminated NEDD4-mediated suppression of PEDV infection (Fig. 3C). To investigate the functional impact of the C2 single-exon deletion in NEDD4, we performed structural modeling of porcine NEDD4 isoform X3 and its C2-deleted variant using AlphaFold3. Visual inspection revealed significant conformational differences between wild-type NEDD4 and the C2-deletion mutant (Fig. 3D and E). Subsequent structural alignment in PyMOL yielded an RMSD value of 4.763, indicating substantial structural divergence (Fig. 3F). Taken together, these results demonstrate that the C2 exon sequence RDDFLGQVDVPLYPLPT is essential for maintaining NEDD4’s structural integrity and its antiviral function against PEDV.

### NEDD4 interacts with NSP8

**Fig 4:**
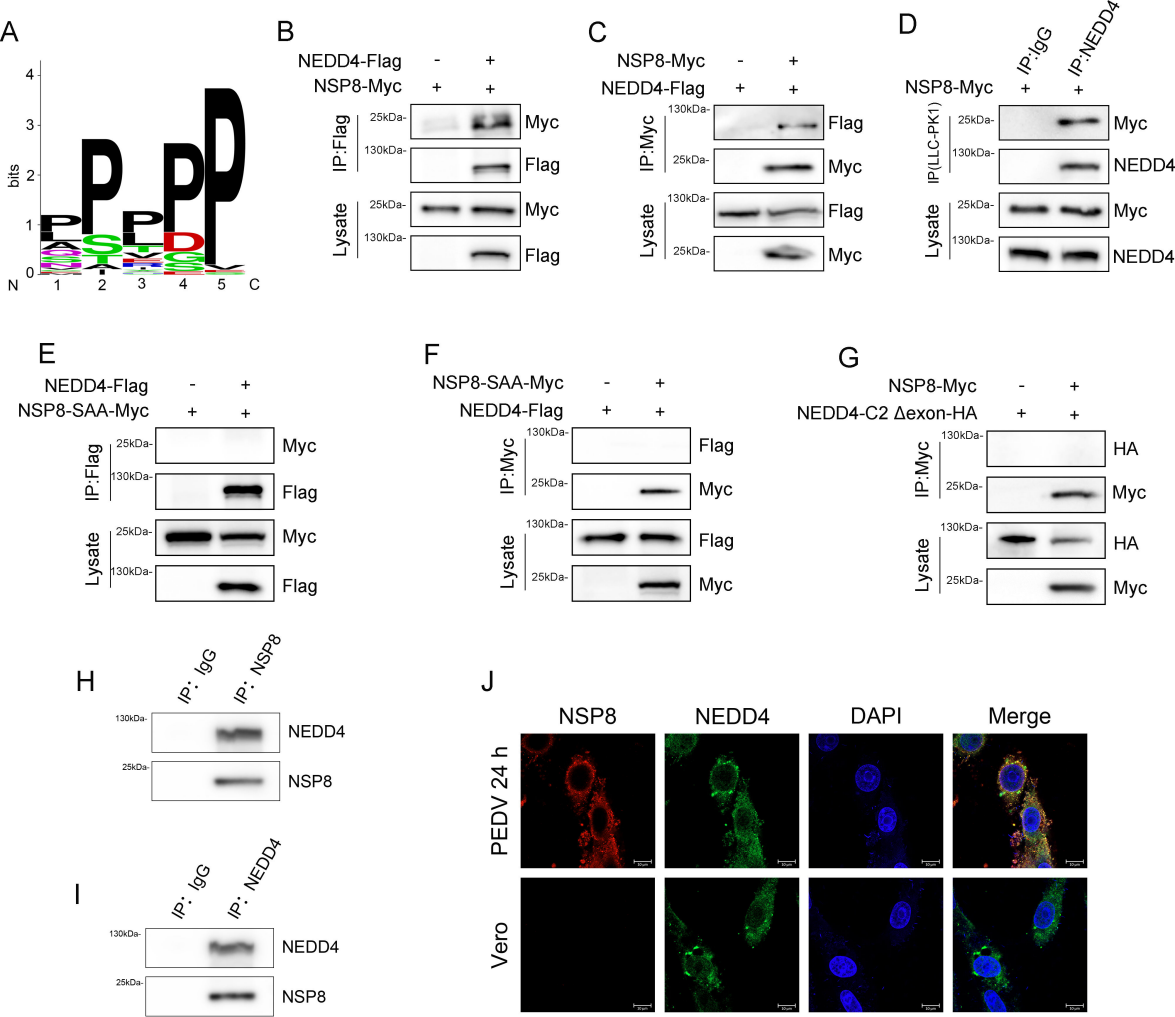
NEDD4 interacts with NSP8. (**A**) Weblogo characterization of ubiquitination site motifs in all NEDD4 substrates identified by UbiNet 2.0. (**B and C**) Immunoblot analysis of Co-IP samples for NSP8 and NEDD4 from HEK-293T cells at 24 h post-overexpression. (**D**) Immunoblot analysis of Co-IP samples for NSP8 and NEDD4 from LLC-PK1 cells at 24 h post-overexpression. (**E and F**) Immunoblot analysis of Co-IP samples for NSP8-SAA and NEDD4 from HEK-293T cells at 24 h post-overexpression. (G) Immunoblot analysis of Co-IP samples for NSP8 and NEDD4 C2 deletion from HEK-293T cells at 24 h post-overexpression. (**H and I**) Immunoblot analysis to detect the interaction between viral NSP8 and host NEDD4 via co-immunoprecipitation in Vero cells at 24 h post-PEDV infection. (**J**) Immunofluorescence assay was performed to examine the localization and potential co-localization of viral NSP8 and host protein NEDD4 in Vero cells at 24 h after PEDV infection.

Having established NEDD4 as a negative regulator of PEDV infection, we next investigated the underlying molecular mechanisms. As an E3 ubiquitin ligase, the most plausible mechanism involves direct ubiquitination-mediated degradation of viral proteins to suppress infection. Given that substrates recognized by E3 ubiquitin ligases typically share conserved features, we systematically analyzed all PEDV-encoded proteins against the known substrate characteristics of NEDD4 provided by UbiNet 2.0 (Fig. 4A) (Table S1) (22). Our screening identified a similar motif SPP within the NSP8 protein, suggesting NSP8 as a potential substrate of NEDD4. Subsequently, HEK-293T cells were transfected with NEDD4 and NSP8 overexpression plasmids either individually or in combination. Reciprocal co-immunoprecipitation (Co-IP) followed by immunoblotting confirmed an interaction between NEDD4 and NSP8 (Fig. 4B and C). To further examine whether endogenous porcine NEDD4 interacts with NSP8, NSP8 was transfected into LLC-PK1 cells, and Co-IP assays were performed with anti-NEDD4 antibody or IgG. Consistent with previous results, NSP8 interacted with porcine NEDD4 in LLC-PK1 cells (Fig. 4D). Subsequently, to verify whether the SPP motif is required for the interaction between NEDD4 and NSP8, we introduced an SAA mutation at the SPP site of NSP8. Co-IP results demonstrated that the PP-AA mutation completely abolished the binding capacity of NEDD4 to NSP8, indicating that the SPP motif is essential for their interaction (Fig. 4E and F). We next examined the interaction between the C2 domain-truncated NEDD4 and NSP8. Results demonstrated that partial deletion of the C2 domain completely abolished NEDD4’s binding capacity to NSP8 (Fig. 4G).

To investigate whether NSP8 interacts with NEDD4 during PEDV infection, polyclonal antibodies against PEDV NSP8 were prepared. At 24 h post-infection of Vero cells with PEDV, Co-IP assays were performed. The results showed that NSP8 could precipitate NEDD4, and conversely, NEDD4 could precipitate NSP8 (Fig. 4H and I). Furthermore, immunofluorescence assays conducted at the same time point revealed extensive co-localization of NEDD4 and NSP8 in infected cells (Fig. 4J). These results indicate that NEDD4 can bind directly to PEDV NSP8.

### NEDD4 ubiquitinates NSP8

**Fig 5:**
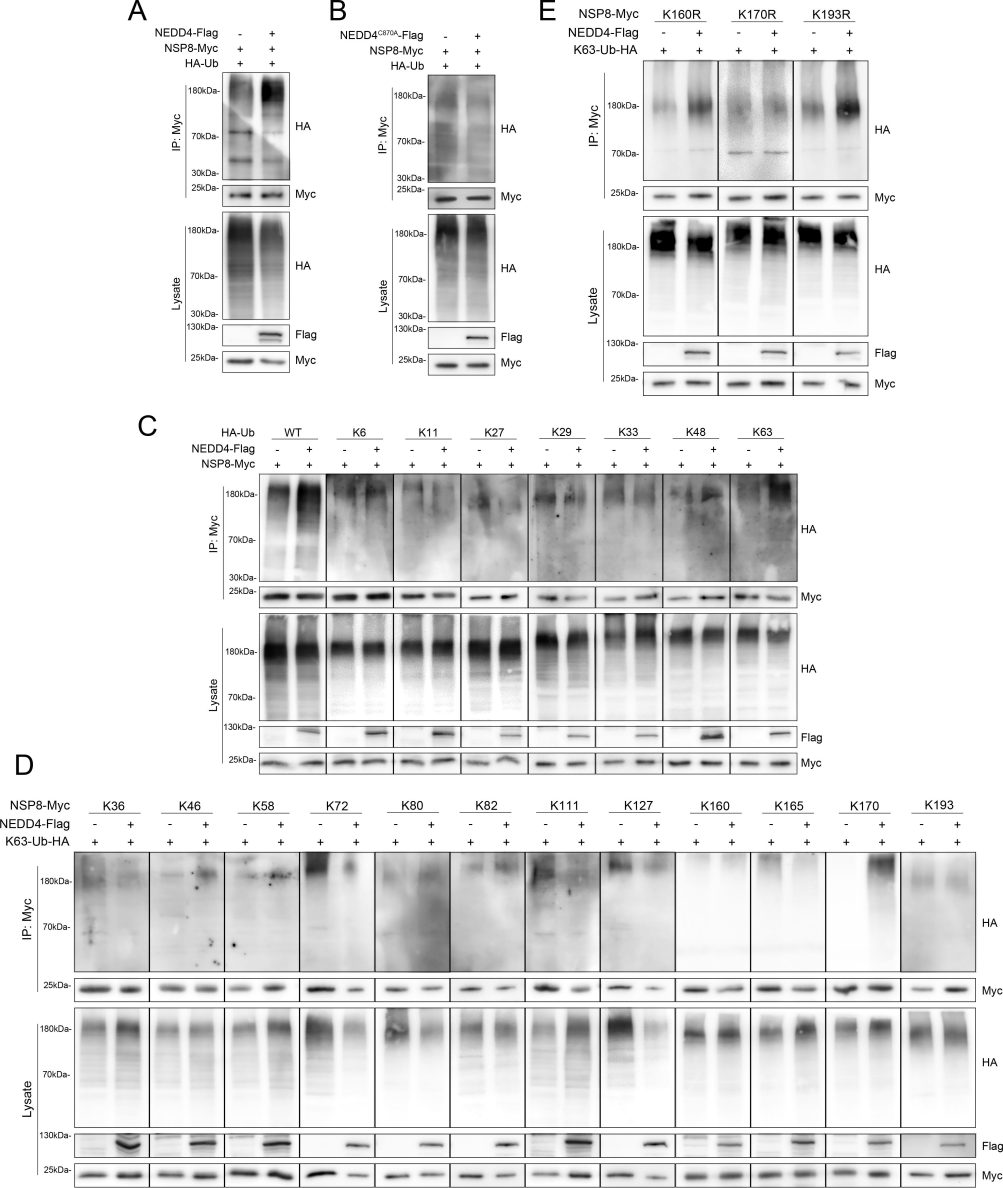
NEDD4 ubiquitinates NSP8. (**A**) The ubiquitination of NSP8 in HEK-293T cells overexpressing NEDD4 and empty vector at 24 h was detected by immunoblotting. (**B**) The ubiquitination of NSP8 in HEK-293T cells overexpressing NEDD4^C870A^ and empty vector at 24 h was detected by immunoblotting. (**C**) The ubiquitination type of NSP8 in HEK-293T cells overexpressing NEDD4 and empty vector at 24 h was detected by immunoblotting. (**D**) The ubiquitination of NSP8 (retaining only a single lysine site) in HEK-293T cells overexpressing NEDD4 and empty vector at 24 h was detected by immunoblotting. (**E**) The ubiquitination of NSP8 (only a single lysine site mutation) in HEK-293T cells overexpressing NEDD4 and empty vector at 24 h was detected by immunoblotting.

As an E3 ubiquitin ligase, NEDD4 catalyzes ubiquitin transfer to its substrates (23). To determine whether NEDD4 mediates ubiquitination of NSP8, HEK-293T cells were co-transfected with NEDD4, NSP8, and ubiquitin overexpression plasmids. Immunoblotting analysis of samples harvested at 24 h post-transfection revealed that NEDD4 overexpression markedly enhanced NSP8 ubiquitination (Fig. 5A). We then constructed the C870A mutant NEDD4 plasmid, which inactivates the HECT domain. Ubiquitination assays revealed that the C870A mutation did not affect NSP8 ubiquitination (Fig. 5B). A series of HA-tagged ubiquitin mutants (K6, K11, K27, K29, K33, K48, and K63), in which only one of the seven lysine residues was reserved and the other lysine residues were mutated, was used to identify the lysine residue in ubiquitin that was required for NEDD4-catalyzed polymeric ubiquitin chain formation. There was only one mutant, K63, which could be linked to NSP8 as efficiently as wild-type ubiquitin (Fig. 5C). To determine which lysine residue in NSP8 is required for NEDD4-catalyzed K63-linked ubiquitination, a series of Myc-tagged NSP8 mutants (K36, K46, K58, K72, K80, K82, K111, K127, K160, K165, K170, and K193), in which only one of the 12 lysine residues was reserved and the other lysine residues were mutated. Mutational analysis identified lysine 170 as the critical residue in NSP8 essential for NEDD4-mediated ubiquitination (Fig. 5D). Another form of NSP8 mutants, K160R, K170R, and K193R, in which one of the 12 lysine residues was mutated, was used to reconfirm the lysine residue in NSP8 that was essential for NEDD4-catalyzed K63-linked ubiquitination. Site-directed mutagenesis demonstrated that the K170 mutation significantly impaired NEDD4-mediated ubiquitination of NSP8 (Fig. 5E). Collectively, NEDD4 mediates K63-linked ubiquitination of PEDV NSP8 at lysine 170.

### NDP52-mediated selective autophagy coordinates with UPS to orchestrate NSP8 degradation

**Fig 6:**
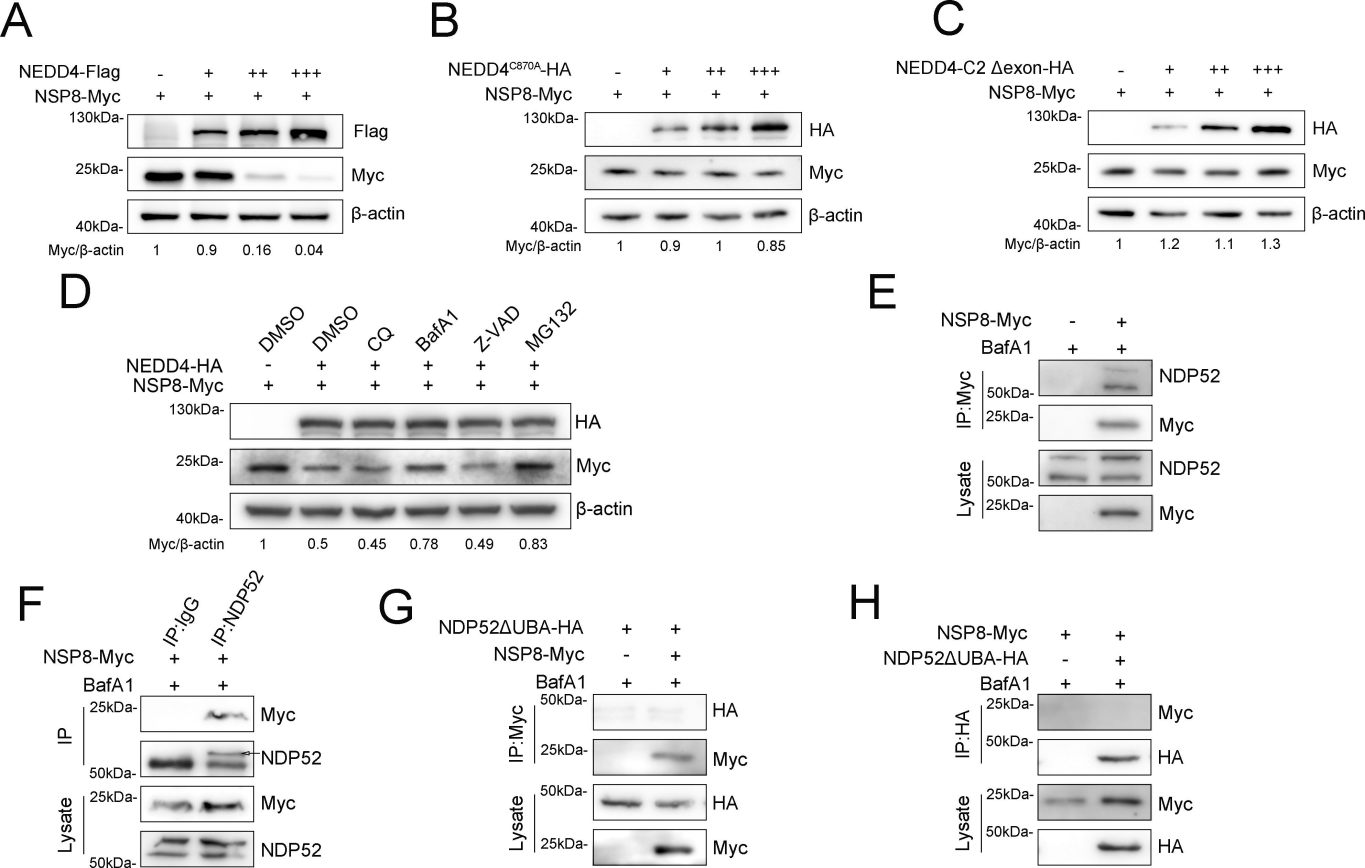
NDP52-mediated selective autophagy cooperates with UPS to degrade NSP8. (**A**) Immunoblot analysis of NSP8 expression in HEK-293T cells with gradient overexpression of NEDD4 for 24 h. (**B**) Immunoblot analysis of NSP8 expression in HEK-293T cells with gradient overexpression of NEDD4^C870A^ for 24 h. (**C**) Immunoblot analysis of NSP8 expression in HEK-293T cells with gradient overexpression of NEDD4 C2 deletion for 24 h. (**D**) Immunoblot analysis of NSP8 expression in HEK-293T cells after 24 h of NSP8 overexpression, followed by 6-h treatment with CQ (30 μM), BafA1 (10 μM), Z-VAD (30 μM), and MG132 (30 μM). (**E and F**) Co-IP analysis of NSP8 and NDP52 in HEK-293T cells overexpressing NSP8 for 24 h followed by BafA1 treatment for 6 h. (**G and H**) Co-IP analysis of NSP8 and NDP52ΔUBA in HEK-293T cells overexpressing NSP8 and NDP52ΔUBA for 24 h followed by BafA1 treatment for 6 h.

Having confirmed NEDD4-mediated ubiquitination of NSP8, we next sought to determine whether this modification regulates NSP8 function or targets it for degradation. HEK-293T cells were co-transfected with NSP8 and increasing doses of NEDD4 plasmid. Immunoblotting analysis at 24 h post-transfection showed that NSP8 protein levels decreased in a NEDD4 dose-dependent manner, suggesting ubiquitin-mediated degradation (Fig. 6A). To further validate this finding, we employed the C870A catalytic mutant of NEDD4. Immunoblotting analysis confirmed that the inactive NEDD4 failed to reduce NSP8 protein levels (Fig. 6B). We next assessed the effect of C2 domain-truncated NEDD4 on NSP8 expression levels. Strikingly, partial deletion of the C2 domain completely abrogated NEDD4’s ability to modulate NSP8 expression (Fig. 6C). To determine which degradation pathway is responsible for the degradation of ubiquitinated NSP8, HEK-293T cells were treated with the proteasome pathway inhibitor MG132, the apoptosis pathway inhibitor Z-VAD, or the autophagy pathway inhibitor CQ and BafA1 for 6 h after co-transfected NEDD4 with NSP8 for 24 h. The results revealed that the NEDD4-mediated degradation of NSP8 was reversed by treatment with MG132 or BafA1 but not Z-VAD or CQ, suggesting that the autolysosome and proteasome are involved in NEDD4-mediated degradation of NSP8 (Fig. 6D). We then confirmed which selective autophagy receptor is responsible for autophagic degradation of NSP8. Accumulating evidence supports that SQSTM1 delivers viral proteins to autophagosomes for selective degradation during infection (24–27). Hence, we first investigated the relationship between SQSTM1 and NSP8 by Co-IP with anti-Myc magnetic beads under treatment with BafA1. However, there was no interaction between SQSTM1 and NSP8 (data not shown). It has been reported that autophagy receptor NDP52 acts as a “bridge” between autophagy and the UPS, and it also plays an important role in the process of selective autophagy (28, 29). Following transfection of Myc-tagged NSP8 into HEK-293T cells, we performed Co-IP using anti-Myc magnetic beads. The result showed that NDP52 interacted with NSP8 (Fig. 6E). Subsequently, reverse Co-IP reconfirmed this result (Fig. 6F). Selective autophagy receptors utilize the UBA domain to bind to ubiquitinated substrates and the LIR domain to interact with LC3 (30, 31). Therefore, we constructed the mutant NDP52ΔUBA-HA to confirm whether NDP52 interacts with NSP8 through the UBA domain. Co-IP assay showed that the deletion of the UBA domain eliminated this interaction (Fig. 6G and H). Thus, the results described above demonstrated that NDP52 acts as a cargo receptor linking NSP8 to the autophagosome.

### NEDD4 upregulates cellular autophagy to enhance its anti-PEDV activity

**Fig 7:**
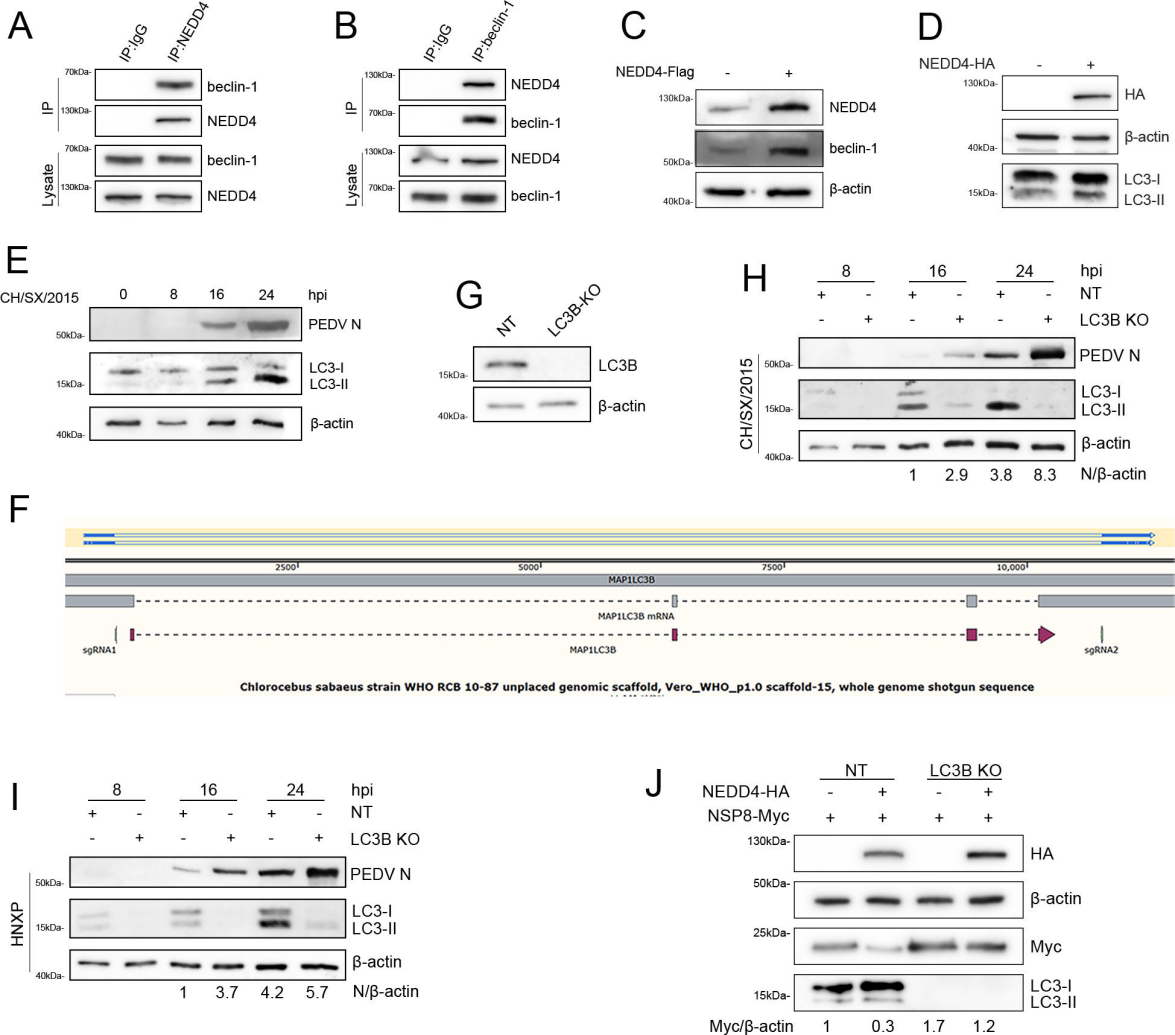
NEDD4 induces autophagy to inhibit PEDV infection. (**A and B**) Co-IP analysis of the interactions between beclin-1 and NEDD4 in Vero cells. (**C and D**) Immunoblot analysis of beclin-1 and LC3B expression in Vero cells following 24-h NEDD4 overexpression. (**E**) Immunoblot analysis of LC3B expression in Vero cells at different time points after PEDV infection. (**F**) Genomic sequencing verification of LC3B KO through gene amplification. (**G**) Immunoblot validation of LC3B KO. (**H and I**) Immunoblot analysis of N protein expression from PEDV classical strain (CH/SX/2015) and epidemic strain (HNXP) in LC3B KO versus NT Vero cells at different time points. (**J**) Immunoblot analysis of NSP8 and NEDD4 expression in LC3B knockout (KO) and control Vero (NT) cells at 24 h post-transfection.

The NDP52-selective autophagic pathway targets NEDD4-ubiquitinated NSP8 for degradation, thereby restricting viral propagation. Intriguingly, NEDD4 has been reported to function as a positive autophagy regulator (32, 33), implying a dual antiviral mechanism where NEDD4 both marks viral components for destruction and potentiates the autophagic machinery itself. To validate the reported pro-autophagic role of NEDD4 (34), we confirmed its interaction with beclin-1 and ability to upregulate beclin-1 expression (Fig. 7A through C). Furthermore, NEDD4 overexpression increased LC3B-II levels, a canonical autophagy marker, demonstrating its positive regulation of autophagic flux (Fig. 7D). Subsequently, the impact of PEDV infection on cellular autophagy was examined, and the results demonstrated that PEDV infection significantly induced the occurrence of autophagy (Fig. 7E). To further clarify the impact of autophagy on PEDV infection, sgRNAs targeting the LC3B gene were designed. After single-cell subcloning screening and sequencing validation, an LC3B knockout cell line was successfully generated (Fig. 7F). Immunoblotting analysis further confirmed the successful knockout of LC3B (Fig. 7G). Next, the LC3B knockout cell line was used to evaluate the impact of autophagy deficiency on PEDV infection. The results showed that the loss of LC3B significantly enhanced the expression of the PEDV N protein, suggesting that autophagy suppresses PEDV infection (Fig. 7H and I). Subsequently, we analyzed the effect of LC3B knockout on NEDD4-mediated expression of NSP8. In LC3B-knockout cell lines and control Vero cells, NSP8 was co-transfected with either an empty vector or NEDD4. The results showed that 24 h after transfection, the protein level of NSP8 was higher in LC3B-knockout cells compared with control cells, and the inhibitory effect of NEDD4 on NSP8 expression was attenuated (Fig. 7J). These findings indicate that NEDD4-mediated degradation of NSP8 depends on the cellular autophagy pathway. Taken together, these results demonstrate that in addition to directly mediating ubiquitination and degradation of PEDV NSP8, NEDD4 can further suppress PEDV infection by positively regulating cellular autophagy.

### NEDD4 mediates the ubiquitination and degradation of alpha coronavirus NSP8

**Fig 8:**
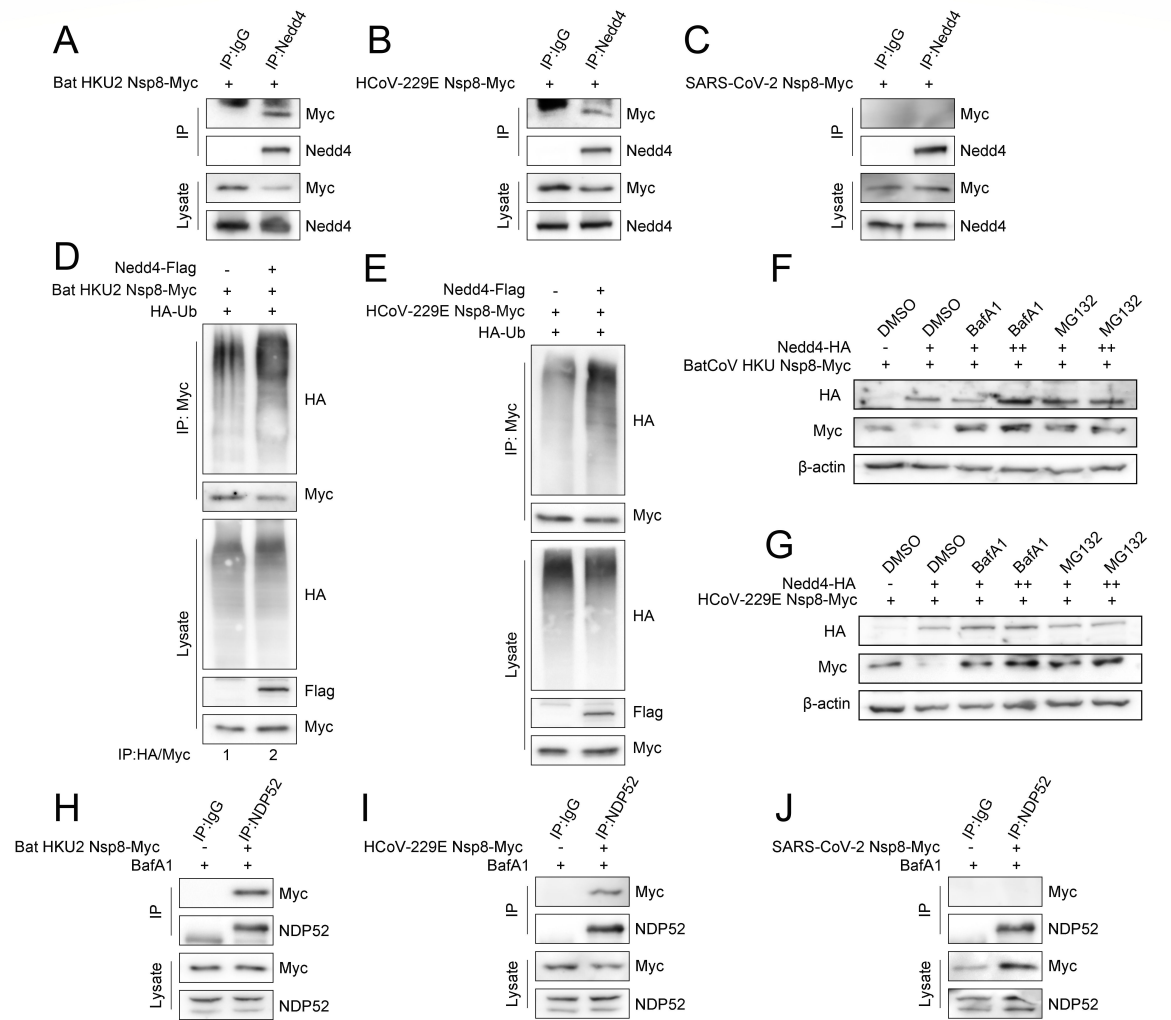
NEDD4 ubiquitinates alpha coronavirus NSP8 and degrades it by UPS and NDP52-mediated selective autophagy. (**A–C**) Co-IP analysis of the interactions between NSP8 (from bat HKU2, HCoV-229E, and SARS-CoV-2) and NEDD4 in HEK-293T cells after 24-h overexpression. (**D and E**) The ubiquitination of bat HKU2 and HCoV-229E NSP8 in HEK-293T cells overexpressing NEDD4 and empty vector at 24 h was detected by immunoblotting. (**F and G**) Immunoblot analysis of bat HKU2 and HCoV-229E NSP8 expression in HEK-293T cells after 24 h of NSP8 overexpression, followed by 6 h treatment with BafA1 (10 or 20 μM) and MG132 (30 or 60 μM). (**H–J**) Co-IP analysis of the interactions between NSP8 (from bat HKU2, HCoV-229E, and SARS-CoV-2) and NDP52 in HEK-293T cells after 24-h overexpression, followed by 6-h treatment with BafA1.

As one member of the RTC for viral replication, NSP8 has a relatively stable tertiary structure, although they share large differences in amino acid sequence among coronavirus groups. To maintain its normal function, the core sequence of NSP8 often exhibits strong conservation during viral evolution (5, 35). Subsequently, we analyzed the conservation of the K170 site and found it to be highly conserved across the entire alpha coronavirus genus, suggesting that the K170 site is crucial for alpha coronavirus NSP8 (Fig. S1A). Subsequently, based on phylogenetic analysis, we selected two representative strains from the same genus as PEDV for further investigation: HCoV-229E NSP8 (relatively closely related) and Bat HKU2 NSP8 (more distantly related) (Fig. S1B). Co-IP assays were performed with anti-NEDD4 antibodies to confirm whether there are interactions between NEDD4 and bat HKU2 NSP8, HCoV-229E NSP8, or SARS-CoV-2 NSP8. The results indicated that NEDD4 interacts with bat HKU2 NSP8 or HCoV-229E NSP8, not SARS-CoV-2 NSP8 (Fig. 8A through C). We next examined the effect of overexpressing NEDD4 on bat HKU2 NSP8 or HCoV-229E NSP8 ubiquitination. The results demonstrated that NEDD4 overexpression significantly enhanced ubiquitination modification of both Bat HKU2 NSP8 and HCoV-229E NSP8 (Fig. 8D and E). To determine whether selective autophagy and UPS are responsible for the degradation of Bat HKU2 NSP8 or HCoV-229E NSP8, NSP8-transfected HEK-293T cells were treated with MG132 or BafA1. The results revealed that the NEDD4-mediated degradation of NSP8 was reversed and upregulated with the MG132 or BafA1 dose increased (Fig. 8F and G). To verify whether NDP52 plays an essential role in the autophagic degradation of NSP8, we overexpressed NSP8 in HEK-293T cells for 24 h and treated with BafA1 for 6 h. Co-IP assays were performed with anti-NDP52 antibodies, and the results showed that Bat HKU2 NSP8 or HCoV-229E NSP8, not SARS-CoV-2 NSP8, was notably coprecipitated with NDP52 (Fig. 8H through J). Taken together, these results support the idea that NEDD4 plays an essential role in the ubiquitinated degradation of alpha coronavirus NSP8.

## DISCUSSION

Our findings reveal that PEDV infection upregulates NEDD4 expression both *in vivo* (porcine intestinal tissues) and *in vitro* (Vero and LLC-PK1 cells), suggesting a host-driven defensive response (Fig. 1). This aligns with previous reports that NEDD4 regulates intestinal homeostasis (20, 36, 37), which may explain its heightened expression in PEDV-infected gut tissues—the primary site of PEDV replication. Further research on the relationship between NEDD4 and PEDV based on intestinal homeostasis will provide us with a more comprehensive understanding of NEDD4’s role in PEDV infection. Additionally, studying intestinal homeostasis may open up new avenues for better controlling PEDV infections.

NEDD4 regulates several cellular processes, including cargo sorting, intracellular trafficking, and degradation of a large number of proteins in multiple cellular compartments by the ubiquitination of its specific substrates (23), suggesting NEDD4 may participate in PEDV infection through multiple pathways. Our experiments confirmed that NEDD4 plays an antiviral role during PEDV infection (Fig. 2). Notably, during the knockout experiments of NEDD4, we observed an unexpected compensatory mechanism: when sgRNAs targeted exon-intron boundaries, the disrupted splicing signals led the cells to skip the targeted exon and splice the remaining exons into a full-length mRNA. Although this spliced variant allowed mRNA expression, it produced a dysfunctional protein. In response, cells further upregulated the expression of this aberrant transcript, reflecting an intrinsic adaptive effort to maintain protein output—a form of cellular self-repair in the face of genetic disruption, albeit one that failed to restore normal function. This compensatory upregulation underscores the complexity of interpreting knockout phenotypes and highlights the cell’s capacity to activate feedback mechanisms upon sensing functional loss. Overall, these results are consistent with studies showing that E3 ubiquitin ligases can restrict viral infections by targeting viral proteins for degradation (38). However, studies have shown that numerous viruses have the ability to exploit NEDD4 to enhance their own infection. These viruses primarily utilize ubiquitination modification to regulate the transport of their structural proteins, thereby promoting viral assembly and budding processes (39, 40). This even includes SARS-CoV-2(19), which belongs to the same species as PEDV. Hence, we also investigated whether PEDV employs a similar NEDD4-dependent mechanism as SARS-CoV-2, but found that the PEDV spike protein lacks the ability to bind NEDD4, which means that, unlike SARS-CoV-2, PEDV cannot utilize NEDD4-mediated ubiquitination of its S protein to facilitate viral budding (data not shown).

NSP8, as a critical component of the coronavirus replication-transcription complex (RTC), plays an essential role in viral RNA synthesis and has been shown to interact with multiple host factors (41). Our study reveals that NSP8 is a key target of the host antiviral protein NEDD4, which mediates its K63-linked ubiquitination and subsequent degradation through both the proteasome and autophagy pathways (Fig. 5 and 6). This finding is particularly significant because NSP8 is highly conserved among alpha coronaviruses, including PEDV, HCoV-229E, and bat HKU2, but not in beta coronaviruses like SARS-CoV-2 (Fig. 8). This host antiviral mechanism broadens its range of antiviral activity. However, members of other coronavirus subgenera have evolved in a different direction to escape this host antiviral mechanism, which demonstrates that viruses and hosts are engaged in a mutual struggle and co-evolution (42, 43). As one of the core components of coronaviruses, NSP8 must maintain functional stability during evolution. While mutations in its core sequence typically carry significant risks, NSP8 has managed to preserve its functionality despite these changes throughout its prolonged evolutionary arms race with the host. This remarkable balance between mutation and functional conservation has conferred a substantial survival advantage to the coronavirus family.

Moreover, NEDD4 exhibits remarkable functional versatility by catalyzing distinct types of ubiquitin modifications that dictate diverse fates of its substrates. While K11-, K29-, and K33-linked ubiquitination by NEDD4 remains unreported, this ligase has been shown to utilize multiple other linkage types to regulate substrate stability, activity, or degradation. Notably, K6- and K27-linked ubiquitination by NEDD4 stabilizes beclin 1 (promoting autophagy) and RORgammat (enhancing Th17-mediated autoimmunity), respectively (34, 44, 45). In this study, we also confirmed that NEDD4 stabilizes beclin 1 (Fig. 7). In contrast, K48-linked polyubiquitination mediates the degradation of various substrates, including TAK1 (46), VDAC1 (47), HBV X (18), and caspase-11 (48). Intriguingly, NEDD4 can employ both K48 and K63 linkages to degrade IGPR-1 and RAP2A, suggesting context-dependent substrate regulation (49, 50). Moreover, K63-linked ubiquitination exerts dual effects: it stabilizes MDM2 and VHL while promoting the autophagic degradation of α-synuclein and RTP801 (51–54). Additionally, NEDD4-mediated K63 ubiquitination of TRAF3 and AKT enhances AKT signaling, further underscoring its role in modulating cellular pathways (55, 56). Our finding that NEDD4 catalyzes K63-linked ubiquitination of PEDV NSP8 for degradation aligns with its broader ability to employ this linkage type for both stabilizing and destabilizing substrates, depending on cellular context. Using inhibitor treatment, we discovered that ubiquitinated NSP8 is degraded via the proteasome pathway and the autophagy pathway (Fig. 6D). Classically, K48-linked chains primarily mediate proteasomal degradation, whereas K63-linked chains are mainly involved in signal transduction and autophagy. However, successive studies have found that K63-mediated ubiquitination can also be recognized and degraded by the proteasome (57, 58). Recent reports have systematically investigated the degradation capacity of the proteasome, revealing that RNP1 acts as a co-receptor for RNP10 to recognize K63-linked and other types of ubiquitination modifications, facilitating their recognition and degradation by the proteasome (59). This further highlights the complexity and inclusiveness of the host system. This functional plasticity positions NEDD4 as a pivotal regulator of viral infection, autophagy, and immune responses through selective ubiquitin code deployment. Future studies should explore whether viral proteins exploit specific NEDD4-mediated ubiquitination types to evade host defenses or promote infection.

The multifaceted role of NEDD4 in antiviral defense is further exemplified by its ability to form complexes with deubiquitinating enzymes, enabling rapid modulation of substrate ubiquitination status in response to cellular demands. Recent studies have demonstrated that NEDD4 cooperates with the deubiquitinase USP13 to remove ubiquitin chains from VPS34A, thereby stabilizing this key autophagy regulator (33). This mechanism synergizes with NEDD4-mediated K6/K27-linked ubiquitination of beclin 1 (44), collectively enhancing autophagic flux. In the context of PEDV infection, this dual regulatory capacity of NEDD4—through both ubiquitination and deubiquitination—creates a robust antiviral environment: while K63-linked ubiquitination marks viral NSP8 for degradation, the NEDD4-USP13-VPS34A/NEDD4-beclin-1 axis simultaneously amplifies autophagic activity to further suppress viral replication. This sophisticated interplay between ubiquitin conjugation and removal highlights the dynamic nature of host antiviral defenses, where NEDD4 serves as a central node coordinating multiple arms of the proteostasis network. However, an important unanswered question remains whether viruses have evolved countermeasures to hijack this ubiquitin-deubiquitinase switch for their benefit, similar to how some viruses exploit host ubiquitin machinery. Future investigations should explore potential viral factors that might interact with or modulate the NEDD4-deubiquitinating enzyme complexes, which could reveal novel mechanisms of viral immune evasion and provide targets for therapeutic intervention. The discovery of such viral strategies would significantly advance our understanding of host-pathogen conflicts at the ubiquitin interface.

**Fig 9:**
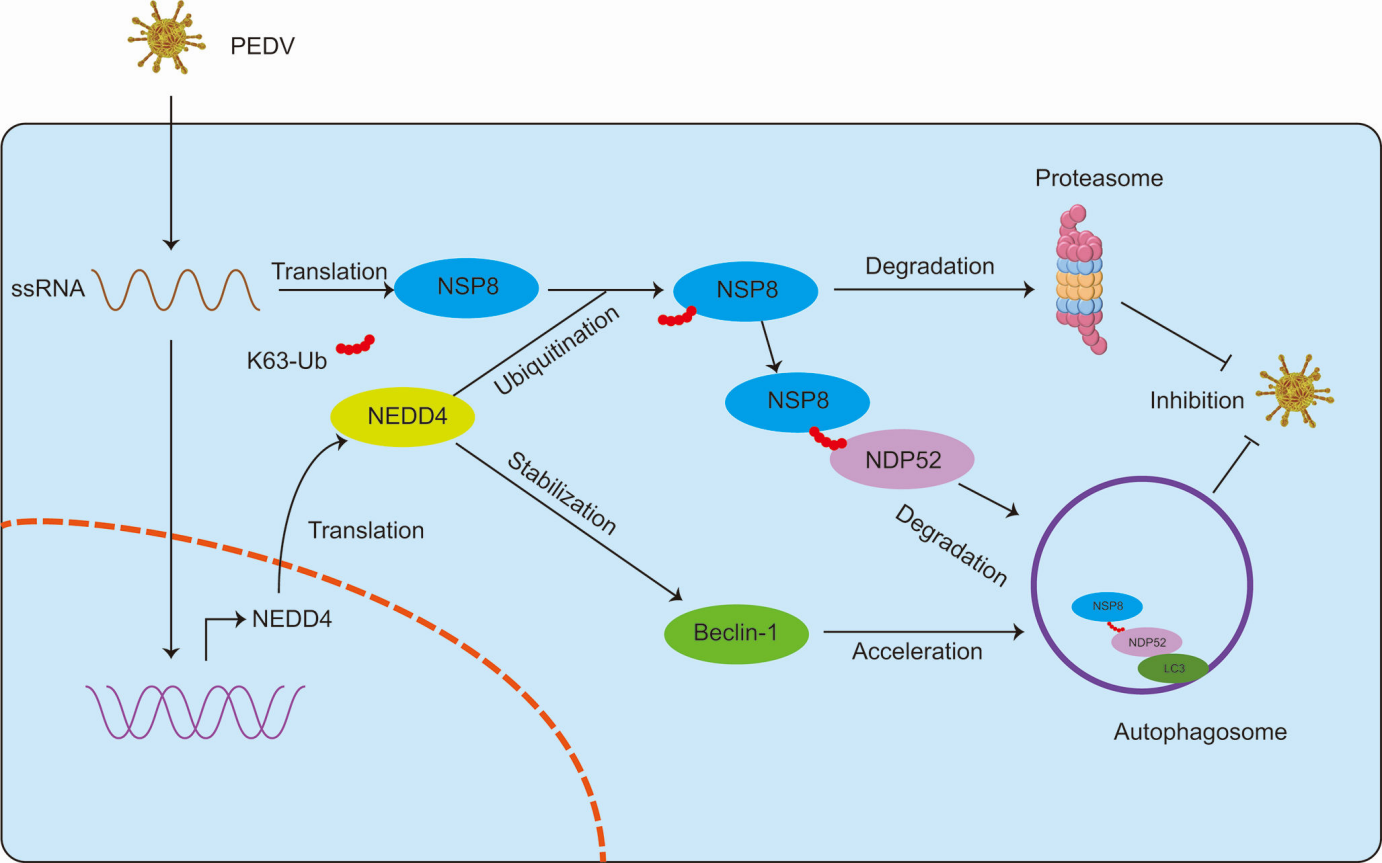
Mechanism diagram of NEDD4 inhibition of PEDV infection. On one hand, NEDD4 inhibits viral replication by ubiquitinating viral NSP8, triggering its degradation via the UPS and NDP52-mediated selective autophagy. On the other hand, NEDD4 enhances autophagy by stabilizing beclin-1 expression, thereby accelerating NSP8 degradation and further suppressing viral infection.

In conclusion, we show that NEDD4, an E3 ubiquitin ligase, is upregulated during PEDV infection and plays an antiviral role by ubiquitinating PEDV NSP8 and promoting its degradation via both the UPS and NDP52-mediated selective autophagy. Furthermore, we show that NEDD4 enhances cellular autophagy, providing an additional layer of antiviral defense. Importantly, this mechanism extends to other alphacoronaviruses, suggesting a conserved antiviral role of NEDD4 in this viral genus (Fig. 9). This discovery not only expands our understanding of host antiviral defense mechanisms but also provides a theoretical foundation for developing novel antiviral strategies targeting viral protein stability.

## MATERIALS AND METHODS

### Antibodies and reagents

The antibodies were commercially available and used according to the manufacturer’s instructions. Antibodies anti-NEDD4 (CY8238, WB 1:2,000; IP 1:200) and anti-Myc tag mouse monoclonal antibody (AB0001; WB 1:2,000) were purchased from Abways. Antibodies anti-β-actin (HC201-02; WB 1:2,000), anti-HA tag mouse monoclonal antibody (HT301-02; WB 1:2,000), anti-GAPDH mouse monoclonal antibody (HC301-01; WB 1:2,000), and anti-DYKDDDDK tag mouse monoclonal antibody (HT201-02; WB 1:2,000) were purchased from TransGen Biotech. Antibodies anti-beclin-1 polyclonal antibody (11306-1-AP, 66665-1-Ig; WB 1:2,000; IP 1:200), anti-NEDD4 (21698, 67865, WB 1:2,000; IP 1:200), anti-NDP52 polyclonal antibody (12229-1-AP; WB 1:2,000; IP 1:200), anti-HA tag polyclonal antibody (51064-2-AP; WB 1:1,000), and anti-DYKDDDDK tag polyclonal antibody (20543-1-AP; WB 1:1,000) were purchased from Proteintech Group. Antibody anti-c-Myc (T55150; WB 1:2,000) was purchased from Abmart. Antibody against PEDV NSP8 was produced by AtaGenix Company. Antibody anti-PEDV N was made in our laboratory. Antibodies Goat anti-Mouse IgG (H+L) HRP Secondary Antibody (31430; 1:5,000) and Goat anti-Rabbit IgG (H+L) HRP Secondary Antibody (31460; 1:5,000) were purchased from Invitrogen. Chemical reagents were purchased and used without further purification. Chloroquine (30 μM, CQ; HY-17589A), MG132 (30 μM; HY-13259), Bafilomycin A1 (10 μM, Baf A1; HY-100558), and Z-VAD-FMK (30 μM; HY-16658B) were purchased from MedChemExpress. jetPRIME Versatile DNA/siRNA transfection reagent (101000046) was purchased from Polyplus.

### Plasmids

The genes encoding viral proteins were synthesized from the CH/SX/2016 strain (GenBank No. MT787025) (60) by Beijing Tsingke Biotech Co., Ltd. and cloned into the pCMV6-entry vector. Flag-tagged or HA-tagged porcine NEDD4 and NDP52 were constructed by cloning the porcine NEDD4 and human NDP52 ORF into the pcDNA3.1-Flag and pcDNA3.1-HA vectors, respectively. HA-tagged Ub, WT, K6, K11, K27, K29, K33, K48, and K63 were purchased from MIAOLING BIOLOGY. Plasmids with point mutations or truncations were constructed by QuickMutation Site-Directed Mutagenesis Kit (Beyotime; D0206S) or chemically synthesized. All plasmid constructs were confirmed by sequence analysis.

### Cells and viruses

HEK-293T and Vero cells were maintained in our laboratory (60, 61) and cultured in Dulbecco’s modified Eagle medium (DMEM; Gibco/Thermo Fisher Scientific, 12100061) with 10% fetal bovine serum (FBS; TransGen Biotech, FS401-02). LLC-PK1 cells were cultured in medium 199 (M199; BasalMedia, China) with 5% FBS (61, 62). All cells were maintained in the incubator with 5% CO_2_ at 37℃. PEDV strain CH/SX/2015 (GenBank accession number MT783684) and HNXP were grown and titrated in Vero cells. PEDV HNXP strain was kindly provided by Changxu Song, College of Animal Science, South China Agriculture University, Guangzhou, China.

### Preparation of NSP8 polyclonal antibody

The optimized sequence of the NSP8 antigen (SSVDTILNLAKDGVVPLSVIPAVSATKLNIVTSDIDSYNRIQREGCVHYAGTIWNIIDIKDNDGKVVHVKEVTAQNAESLSWPLVLGCERIVKLQ) was cloned into a prokaryotic expression vector with the addition of a 6×His tag. The antigen was purified using nickel affinity chromatography, emulsified with Freund’s adjuvant, and administered to rabbits via subcutaneous injection on the back. Immunization was performed three times at 7-day intervals. Serum antibody titers were measured by ELISA, and following the collection of all sera, the serum antibodies were purified.

### Quantitative reverse transcription PCR

Total RNAs were extracted from cells by use of the RNAiso Plus (Takara; 9109). For the reverse transcription quantitative PCR (RT-qPCR), cDNA was generated with the HiScript II Q Select RT SuperMix for qPCR kit (Vazyme, R232–01) and was amplified by qPCR using ChamQ Universal SYBR qPCR Master Mix (Vazyme, Q711–02) with specific PCR primers (Table 1). The expression levels of genes of interest were normalized to that of the respective internal control actin and presented as fold change relative to the control. The analysis of each gene expression was repeated in at least three independent experiments.

**TABLE 1:** Primers used in this study

Gene	Sequence (5′-3′)
PEDV N	F: GAATTCCCAAGGGCGAAAAT
	R: TTTTCGACAAATTCCGCATCT
β-actin (green monkey)	F: ATCGTGCGTGACATTAAG
	R: ATTGCCAATGGTGATGAC
NEDD4 (green monkey)	F: AACCGAGAGTCTTCCGAGAAC
	R: CACCTGCTGGCTTGAATCAC
β-actin (pig)	F: CCACTGGCATCGTGATGGACTC
	R: CAGCACCGTGTTGGCGTAGA
NEDD4 (pig)	F: TGCCATCAAGCCAGAGTTCT
	R: ATTCTTCCATCGGTGTGAGTTC

### Immunoprecipitation and immunoblot analysis

The experiments were performed as described previously (63). Immunoprecipitation (IP) was performed using HEK-293T cells, LLC-PK1 cells, and Vero cells. The cells were harvested and lysed in RIPA (Solarbio; R0010) containing protease inhibitor PMSF (Solarbio; P0100). Then incubated with the appropriate antibodies plus protein A/G beads (TransGen Biotech; DP501-01), Anti-c-Myc Magnetic Beads (MedChemExpress; HY-K0206), Anti-Flag Magnetic Beads (MedChemExpress; HY-K0207), or Anti-HA Magnetic Beads (MedChemExpress; HY-K0201) overnight. The beads were washed five times with PBST (1× phosphate-buffered saline, 0.1% Tween 20 Detergent), the immunoprecipitations were eluted from the beads with RIPA plus 5× sodium dodecyl sulfate (SDS) loading buffer and resolved on 10% SDS-polyacrylamide gels (SDS-PAGE). Proteins were transferred to polyvinylidene fluoride (PVDF) membranes (millipore/Merck, IPVH09120), followed by blocking with 5% skim milk (Beyotime, P0216) and incubating with the appropriate antibodies, and then the protein bands were visualized by the ECL kit (Advansta, K12045D20).

### Immunofluorescence assay

For immunofluorescence, cells were fixed with 4% paraformaldehyde (PFA) for 15 min, permeabilized with 0.1% Triton X-100 for 10 min, and then blocked with 1% BSA in PBS for 2 h at room temperature. Subsequently, the cells were incubated with the primary antibody for 2 h at 37°C. After three washes with PBS, a fluorescent secondary antibody was applied for 1 h. Nuclei were counterstained with DAPI for 5 min. Images were captured using a Zeiss LSM980 confocal microscope.

### Inhibitor treatment

HEK-293T cells were transfected with plasmids for 24–36 h, and then the culture medium was replaced with fresh medium containing inhibitors and incubated for another 6 h. The total cell lysates were subjected to SDS-PAGE and western blotting. MG132 (30 μM) was used to inhibit proteasome-mediated protein degradation. CQ (30 μM) or bafilomycin A1 (BafA1, 10 μM) was used to inhibit autophagic protein degradation. Z-VAD (30 μM) was used to inhibit the apoptosis pathway.

### CRISPR/Cas9-mediated knockout of NEDD4 in Vero cells

To disrupt the NEDD4 gene, we designed a CRISPR/Cas9 single guide RNA system to target the first common exon or the initiation exon of the HECT domain for the frameshift knockout by using the optimized design software at http://crispr.mit.edu. The 20-nucleotide (nt) guide sequence (First common exon, GCACCTATACCAACCGGTAA; HECT, ACTTCCTCAAGGCTCGACTA; LC3B KO sgRNA1, TCGGTGACGCGCTGCGAGTC; LC3B KO sgRNA2, GGGGTTACGCTTCACAACTC) was cloned into pLentiCRISPRv2 containing a Cas9 expression cassette. Lentivirus particles harboring Cas9/NEDD4 sgRNA were transduced into Vero cells. After selection with 3 mg/mL puromycin, single clones stably expressing sg-NEDD4 were isolated. To identify the status of genome editing, we performed PCR amplification of the genomic DNA isolated from different clonal cell lines by using primers specific to the target sequence and then performed DNA sequencing (61).

### Cell viability assay

The experiments were performed as described previously (61). Briefly, wild-type and NEDD4-knockout Vero cells were seeded in 96-well plates at a density of 5,000 cells per well and cultured for 24 h. Blank control wells containing an equal volume of culture medium without cells were included. Subsequently, 10 μL of CCK8 solution (Solarbio; Cat. No. CA1210) was added to each well. After treatment, the cells were incubated at 37°C in the dark for 2 h, and the absorbance at 450 nm was finally measured using a microplate reader.

### Evolutionary analysis by maximum likelihood method

The evolutionary history was inferred by using the Maximum Likelihood method and the JTT matrix-based model (64). The tree with the highest log likelihood (−1887.40) is shown. The percentage of trees in which the associated taxa clustered together is shown next to the branches. Initial tree(s) for the heuristic search were obtained automatically by applying Neighbor-Join and BioNJ algorithms to a matrix of pairwise distances estimated using the JTT model, and then selecting the topology with the superior log likelihood value. The tree is drawn to scale, with branch lengths measured in the number of substitutions per site. This analysis involved nine amino acid sequences. There was a total of 198 positions in the final data set. Evolutionary analyses were conducted in MEGA11 (65).

### Statistical analyses

Grayscale scanning was performed using ImageJ and normalized using β-actin as the reference. Data were represented as mean ± SD, and Student’s t-test was used for all statistical analyses with the GraphPad Prism 9 software. Differences between the two groups were considered significant when the *P* value was less than 0.05.

## Data Availability

All data are contained within the article.

## SUPPLEMENTAL MATERIAL

Fig. S1 jvi.02156-25-s0001.doc K170 in Nsp8 is conserved among alpha coronavirus.

Table S1 jvi.02156-25-s0002.xlsx Motif information recognized by NEDD4.

ASM does not own the copyrights to Supplemental Material that may be linked to, or accessed through, an article. The authors have granted ASM a non-exclusive, world-wide license to publish the Supplemental Material files. Please contact the corresponding author directly for reuse.
