# Supplementary material for: E3 ubiquitin ligase NEDD4 inhibits PEDV infection through ubiquitination and degradation of the viral primase NSP8
Source: J Virol. 2026 Mar 30;100(4):e02156-25. doi: 10.1128/jvi.02156-25 (PMC13098235; doi:10.1128/jvi.02156-25)
Supplement: Fig. S1 — K170 in Nsp8 is conserved among alpha coronavirus. [file jvi.02156-25-s0001.doc]

**Supporting information**


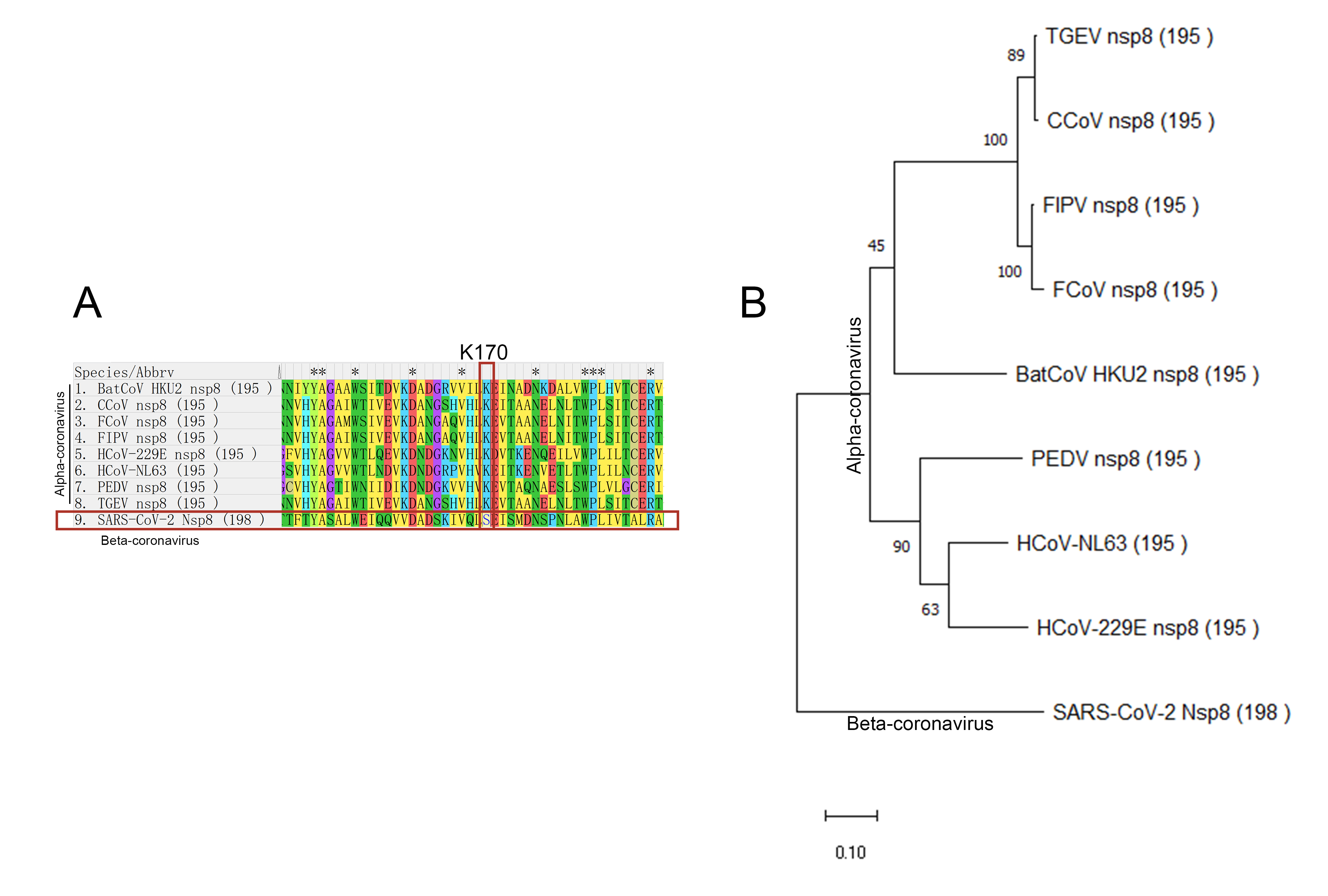


Fig. S1. K170 in Nsp8 is conserved among alpha coronavirus. (A) Analysis and comparation of alpha coronavirus Nsp8 amino acid sequence by software MEGA11. (B) Phylogenetic tree analysis of alpha coronavirus Nsp8 amino acid sequence by software MEGA11.
